# Modulation of Breast Cancer Cell Apoptosis and Macrophage Polarization by Mistletoe Lectin in 2D and 3D Models

**DOI:** 10.3390/ijms25158459

**Published:** 2024-08-02

**Authors:** Chang-Eui Hong, Su-Yun Lyu

**Affiliations:** 1 College of Pharmacy, Sunchon National University, Suncheon 57922, Republic of Korea; gruni80@naver.com; 2 Research Institute of Life and Pharmaceutical Sciences, Sunchon National University, Suncheon 57922, Republic of Korea

**Keywords:** MCF-7, 3D cell culture, M1 macrophage, M2 macrophage, mistletoe lectin

## Abstract

Korean mistletoe (*Viscum album* L. var. *coloratum*) is renowned for its medicinal properties, including anti-cancer and immunoadjuvant effects. This study aimed to elucidate the mechanisms by which Korean mistletoe lectin (*V. album* L. var. *coloratum* agglutinin; VCA) modulates breast cancer cell apoptosis and macrophage polarization. The specific objectives were to (1) investigate the direct effects of VCA on MCF-7 breast cancer cells and THP-1-derived M1/M2 macrophages; (2) analyze the impact of VCA on the paracrine interactions between these cell types; and (3) compare the efficacy of VCA in 2D vs. 3D co-culture models to bridge the gap between in vitro and in vivo studies. We employed both 2D and 3D models, co-culturing human M1/M2 macrophages with human MCF-7 breast cancer cells in a Transwell system. Our research demonstrated that M1 and M2 macrophages significantly influenced the immune and apoptotic responses of breast cancer cells when exposed to VCA. M1 macrophages exhibited cytotoxic characteristics and enhanced VCA-induced apoptosis in both 2D and 3D co-culture models. Conversely, M2 macrophages initially displayed a protective effect by reducing apoptosis in breast cancer cells, but this protective effect was reversed upon exposure to VCA. Furthermore, our findings illustrate VCA’s ability to modulate M1 and M2 polarization in breast cancer cells. Finally, the use of magnetic 3D cell cultures suggests their potential to yield results comparable to conventional 2D cultures, bridging the gap between in vitro and in vivo studies.

## 1. Introduction

Breast cancer represents a significant global health threat, being the most frequent cause of cancer-related deaths in women [1]. It is estimated that 1 in every 8 women will be affected by mammary gland cancers [2], with only 5 to 10% of cases attributed to inherited factors and 90 to 95% linked to environmental causes [3].

The complex interplay among host cells, tumor cells, and various genetic and epigenetic mutations within breast cancers shapes the tumor microenvironment, playing a pivotal role in tumor progression and therapy response [4]. Macrophages constitute the main leukocyte population in breast cancers and are integral to numerous stages of cancer development [5]. These cells can alter their phenotype in response to various environmental triggers, leading to distinct functional states [6,7].

Macrophages can be polarized into two main phenotypes: M1 and M2. M1 macrophages, or classically activated macrophages, exhibit acute inflammatory properties and produce pro-inflammatory cytokines and lymphokines [8]. Key markers of M1 polarization include IL-6, IL-12, TNF-α, and surface markers like CD80 and CD86 [7]. Conversely, M2 macrophages, or alternatively activated macrophages, inhibit inflammatory reactions and facilitate angiogenesis, tissue reconstruction, and healing [9]. M2 polarization results in the generation of anti-inflammatory cytokines, including IL-10, CCL18, and CCL22, and the expression of surface markers such as CD163 and CD206 [10,11].

The balance between M1 and M2 macrophages in the tumor microenvironment significantly influences cancer progression and treatment response. While M1 macrophages generally exhibit anti-tumor properties, M2 macrophages are often associated with tumor promotion and poor prognosis in various cancers, including breast cancer [12].

Tumor-associated macrophages (TAMs) comprise the majority of stromal cells in solid tumors, significantly influencing various aspects of neoplastic tissue [13]. In cancerous tumors, TAMs predominantly exhibit a phenotype similar to M2-activated macrophages, promoting tumor growth, angiogenesis, and metastasis [14]. The interaction between TAMs and cancer cells is complex and bidirectional, creating a supportive microenvironment for tumor growth and contributing to therapy resistance [15].

Given their crucial role in tumor progression, targeting TAMs or reprogramming them towards an M1 phenotype has emerged as a promising approach for cancer treatment [16]. Recent studies have demonstrated that modulating macrophage polarization can affect cancer cell sensitivity to various therapies [17,18]. However, the mechanisms underlying these effects are not fully understood, particularly in the context of natural compounds with anti-cancer properties.

Korean mistletoe (*Viscum album* L. var. *coloratum*) has gained increasing attention due to its potentially superior anti-cancer properties compared to European mistletoe [19]. The primary active component, Korean mistletoe lectin (*Viscum album* L. var. *coloratum* agglutinin, VCA), shows higher cytotoxicity in cancer cells and has demonstrated promise in inhibiting various types of cancer both in vitro and in vivo [20,21]. VCA’s mechanisms of action include the induction of apoptosis, suppression of telomerase activity, and activation of immune cells [22,23].

Despite growing evidence supporting VCA’s cytotoxic and immunological properties, its effects on macrophage polarization and the subsequent influence on breast cancer cells have not been thoroughly investigated. Our study aims to bridge this knowledge gap by examining the complex interactions between VCA, breast cancer cells, and macrophages of different polarization states (M1 and M2).

We employ both 2D and 3D co-culture systems to comprehensively investigate these interactions:

1. Two-dimensional co-culture system: using a Transwell system to study paracrine interactions between MCF-7 breast cancer cells and M1 or M2 macrophages, and how VCA modulates these interactions.
2. Three-dimensional spheroid model: creating co-culture spheroids to better mimic the in vivo tumor microenvironment and study how spatial organization and direct cell–cell contacts influence the response to VCA.

This dual approach allows us to

(a) Investigate VCA’s direct effects on each cell type individually.
(b) Examine VCA’s modulation of paracrine signaling between cancer cells and macrophages.
(c) Assess how the 3D tumor microenvironment influences these interactions.
(d) Evaluate the potential of 3D cultures in bridging the gap between in vitro and in vivo studies.

We hypothesize that VCA will differentially affect breast cancer cells depending on their interactions with M1 or M2 macrophages, potentially reprogramming the tumor microenvironment towards a more anti-tumor state. Our findings may provide new insights into VCA’s potential as an immunomodulatory anti-cancer agent, contributing to the development of novel therapeutic strategies that leverage the complex interactions within the tumor microenvironment to enhance cancer treatment efficacy.

## 2. Results

### 2.1. THP-1 Monocyte Differentiation and Polarization into M1/M2 Macrophages

**Figure 1:**
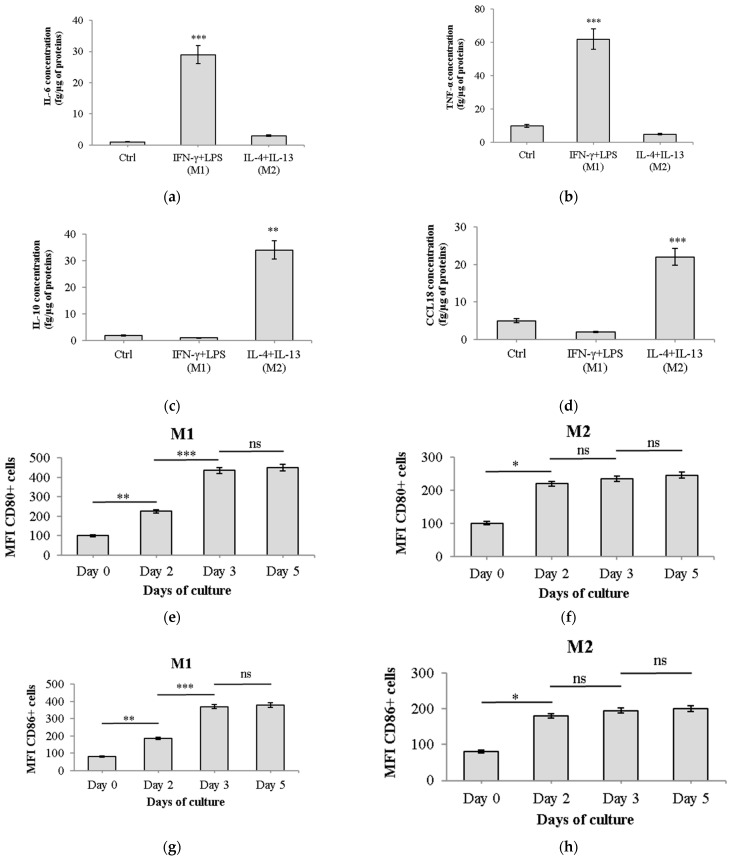

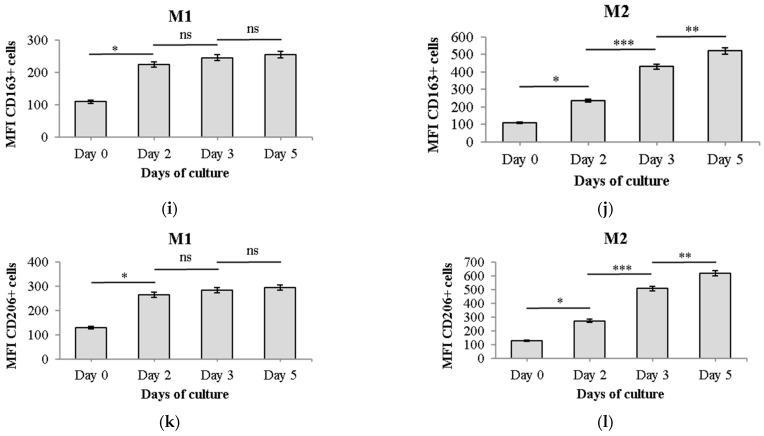
Characterization of M1 and M2 macrophage polarization. THP-1 monocytes were differentiated with PMA for 48 h, then polarized towards M1 or M2 phenotypes. M1 polarization was induced with 20 ng/mL IFN-γ and 100 ng/mL LPS, while M2 polarization was induced with 20 ng/mL IL-4 and 20 ng/mL IL-13. (**a**–**d**) Cytokine secretion measured by ELISA after 24 h (M1) or 72 h (M2) of polarization: (**a**) IL-6, (**b**) TNF-α, (**c**) IL-10, and (**d**) CCL18. Results are expressed as means ± S.D (*n* = 3). (**e**–**l**) Flow cytometry analysis of surface markers during differentiation and polarization: (**e**,**g**) CD80 and CD86 expression in M1-polarized macrophages; (**f**,**h**) CD80 and CD86 expression in M2-polarized macrophages; (**i**,**k**) CD163 and CD206 expression in M1-polarized macrophages; (**j**,**l**) CD163 and CD206 expression in M2-polarized macrophages. Data are presented as mean fluorescence intensity (MFI) ± SEM from three independent experiments. Time points: Day 0 (Undifferentiated THP-1), Day 2 (After 48 h PMA), Day 3 (24 h after polarization stimuli), Day 5 (72 h after polarization stimuli). Statistical significance was determined by one-way ANOVA followed by Tukey’s post hoc test for (**e**–**l**), and by Student’s *t*-test for (**a**–**d**). * *p* < 0.05, ** *p* < 0.01, *** *p* < 0.001, ns: not significant. All experiments were performed in triplicate, with three independent repetitions.

THP-1 human monocytes were incubated with 10 ng/mL of PMA for 48 h, inducing their differentiation into macrophages. After a 3-day resting period, the resulting M0 THP-1 macrophages were polarized into the M1 phenotype by exposure to 20 ng/mL IFN-γ and 100 ng/mL LPS for 24 h. The protein levels of classic M1 markers, TNF-α and IL-6, were measured using ELISA. Incubation of M0 macrophages with IFN-γ and LPS for 24 h led to a significant increase in both cytokines. However, there were no observable changes in their expression profiles. Additionally, the secretion of two M2 markers, IL-10 and CCL18, was measured in M1 macrophages, but no significant alterations were detected (Figure 1a,b).

To induce classical M2 polarization, M0 THP-1 macrophages were incubated with 20 ng/mL IL-4 and 20 ng/mL IL-13 for 72 h. ELISA was then used to measure the protein levels of two markers associated with the M2 state: IL-10 and CCL18. After 72 h of incubation, ELISA confirmed the elevated expression of these cytokines in THP-1-derived M2 macrophages compared to control M0 macrophages. Notably, there were no changes in the secretion of M1 markers (TNF-α and IL-6) in M2-polarized macrophages (Figure 1c,d). To ensure proper macrophage differentiation and polarization, we performed flow cytometry analysis of M1 markers (CD80 and CD86) and M2 markers (CD163 and CD206) at key timepoints during our protocol (Figure 1e–l). We analyzed cells at Day 0 (undifferentiated THP-1), Day 2 (after PMA treatment), Day 3 (24 h after polarization stimuli for M1, 24 h into M2 polarization), and Day 5 (72 h after M2 polarization stimulus).

M1 macrophages showed a progressive increase in CD80 and CD86 expression, reaching peak levels at Day 3 and maintaining them through Day 5 (Figure 1e,g). Statistical analysis showed significant increases in CD80 and CD86 expression in M1 macrophages between Day 0 and Day 3 (*p* < 0.001), with no significant changes between Day 3 and Day 5. The mean fluorescence intensity (MFI) for CD80 increased from 100 ± 5 at Day 0 to 450 ± 17 at Day 5, while CD86 increased from 80 ± 4 to 380 ± 13. In contrast, cells treated with IL-4 + IL-13 showed minimal changes in these markers.

M2 macrophages showed significant increases in CD163 and CD206 expression from Day 0 to Day 5 (*p* < 0.001). M2 macrophages demonstrated a gradual increase in CD163 and CD206 expression, with maximum levels observed at Day 5 (Figure 1j,l). The MFI for CD163 increased from 110 ± 5 at Day 0 to 520 ± 18 at Day 5, and the MFI for CD206 increased from 130 ± 6 to 620 ± 20. Cells treated with IFN-γ + LPS (M1 polarization) showed minimal changes in these M2 markers (Figure 1i,k). Also, while M2-polarized macrophages showed some basal production of TNF-α and IL-6, the levels were significantly lower compared to M1-polarized macrophages (*p* < 0.001).

These results confirm the successful polarization of THP-1 macrophages into distinct M1 and M2 phenotypes, as evidenced by both cytokine production and surface marker expression. The stability of marker expression over time validates the robustness of our differentiation protocol and ensures that the macrophage phenotypes were maintained during the entire course of our experiments.

### 2.2. Effect of VCA on Cell Viability

**Figure 2:**
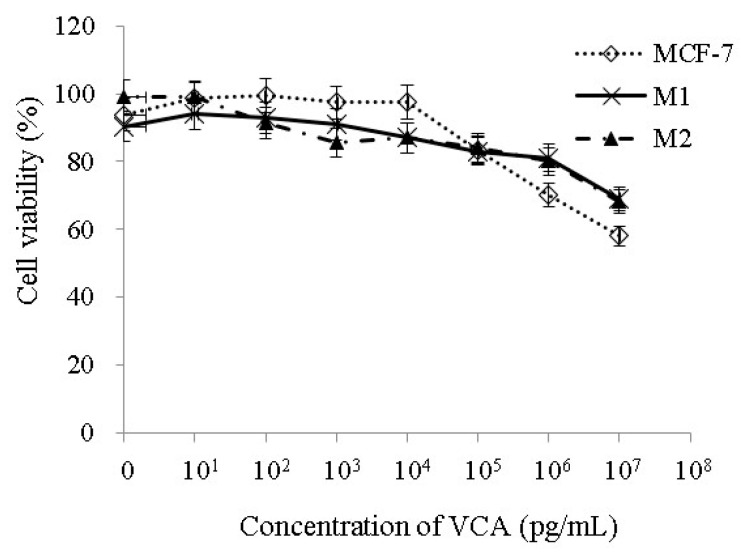
A composite of three independent cell viability tests performed on MCF-7 cells and M1 and M2 macrophages. Cells were cultured in the absence or presence of different concentrations of VCA and the viability was measured by MTT assay. Results are expressed as means ± S.D (*n* = 3). All experiments were performed in triplicate, with three independent repetitions.

To assess the potential cytotoxic effects of VCA on M1 and M2 macrophages, we exposed these cells to varying doses of VCA, ranging from 1 pg/mL to 10 μg/mL, for a duration of 24 h. The results clearly indicated that when exposed to 1 μg/mL of VCA, both M1 and M2 macrophages maintained over 80% viability (Figure 2). Therefore, for our subsequent studies, we selected VCA concentrations of 1 μg/mL and 100 pg/mL due to their minimal impact on M1 and M2 macrophages in comparison to the control. We also applied various concentrations of VCA to MCF-7 cells to assess its effectiveness (Figure 2).

### 2.3. Effect of VCA on M1 and M2 Polarization Marker Secretion in MCF-7 Cells, Co-Cultured with Macrophages

**Figure 3:**
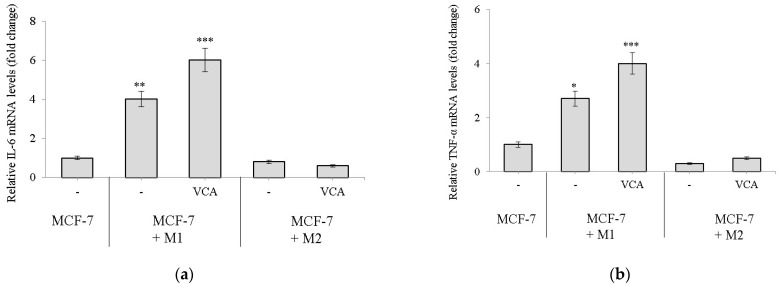

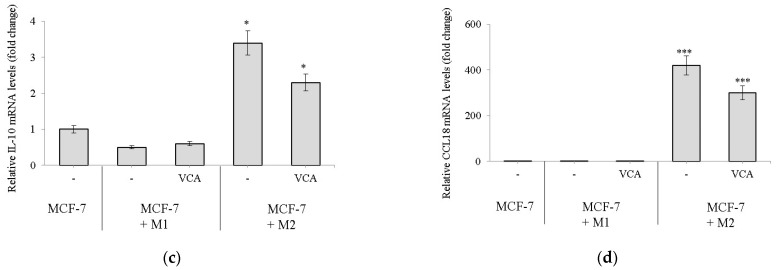
Determination of M1 polarization marker expressions (**a**) IL-6 and (**b**) TNF-alpha, and M2 polarization marker expressions (**c**) IL-10 and (**d**) CCL18 in MCF-7 co-cultured macrophages. M1 and M2 were co-cultured in a Transwell with MCF-7 cells with or without 100 pg/mL of VCA for 24 h. At the end of the incubation, macrophages were gathered and RNA expressions were measured by qPCR. Results are expressed as means ± S.D (*n* = 3). Results are expressed as means ± S.D (*n* = 3). Statistical significance was determined by Student’s *t*-test. * *p* < 0.05, ** *p* < 0.01, *** *p* < 0.001. All experiments were performed in triplicate, with three independent repetitions.

We employed qPCR to assess the expression levels of M1 and M2 polarization markers in macrophages when co-cultured with MCF-7 cells in the presence or absence of 100 pg/mL VCA. Notably, the presence of VCA affected the expressions of M1 markers (IL-6 and TNF-α) in M1 macrophages (Figure 3a,b). As for M2 macrophage markers, IL-10 and CCL18 expressions were reduced in M2 macrophages treated with VCA (Figure 3c,d). This finding is consistent with earlier studies that have reported mistletoe lectin’s ability to enhance TNF-α release while inhibiting IL-10 production [24,25].

### 2.4. Effect of VCA on Apoptosis and Mitochondrial Membrane Potential in MCF-7 Cells, Co-Cultured with Macrophages

**Figure 4:**
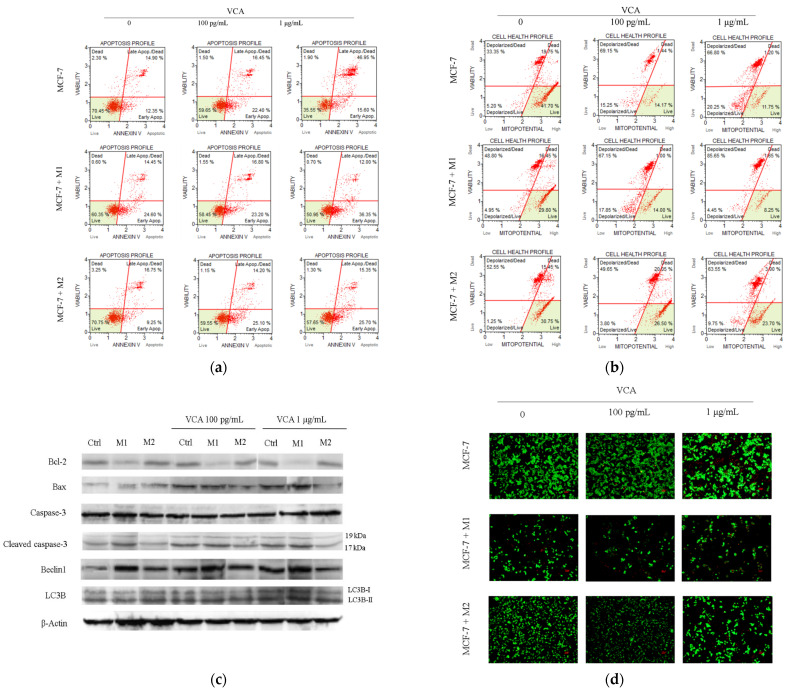
Determination of the (**a**) early and late apoptosis, (**b**) mitochondrial membrane potential, and (**c**) apoptosis-related protein expressions in MCF-7 cells co-cultured with M1 or M2 macrophages when treated with or without VCA for 24 h. (**d**) LIVE/DEAD cell assay was used with acridine orange (AO, excitation 490 nm, emission 515 nm) and propidium iodide (PI, excitation 535 nm, emission 617 nm) after 24 h of cell culture with varying doses of VCA. Scale bar = 100 μm. All experiments were performed in triplicate, with three independent repetitions.

In the co-culture setup with MCF-7 cells, M1 macrophages caused a significant increase in the percentage of apoptotic cells when subjected to VCA treatment compared to controls. In MCF-7 and M1 macrophage co-cultures, the early apoptotic cell population increased from 24.65 ± 0.07% to 35.79 ± 1.77% following administration of 1 ng/mL VCA. In the case of MCF-7 and M2 macrophage co-culture, the early apoptotic cell population rose from 10.71 ± 2.34% in the control to 25.50 ± 0.52% and 26.03 ± 1.79% following treatment with 100 pg and 1 μg/mL VCA, respectively (Figure 4a). This suggests that while M2 macrophages generally provide a protective impact on cancer cells, VCA can counteract this effect and stimulate apoptotic death of cancer cells. To validate the mechanism of VCA-induced apoptosis, we used the MuseTM MitoPotential Kit to observe alterations in mitochondrial membrane potential in MCF-7 cells. In MCF-7 cells co-cultured with M1 macrophages, an increase in the number of depolarized (live and dead) cells was observed after treatment with 100 pg/mL VCA (live: 17.48 ± 1.98% and dead: 68.74 ± 2.72%) and 1 μg/mL VCA (live: 4.45 ± 0.10% and dead: 86.15 ± 2.72%), compared to untreated cells (live: 4.87 ± 0.17% and dead: 47.91 ± 2.97%). In MCF-7 and M2 macrophage co-culture, the population of depolarized cells also increased from 1.22 ± 0.03% (live) and 51.05 ± 2.15% (dead) in the control to 9.25 ± 0.75% and 63.27 ± 2.43% following treatment with 1 μg/mL VCA (Figure 4b).

Furthermore, VCA treatment increased the expressions of apoptosis-related proteins (Bax and cleaved caspase-3) in MCF-7 cells co-cultured with M1 macrophages compared to control cells without VCA incubation. However, when MCF-7 cells were co-cultured with M2 macrophages, these proteins exhibited lower levels under VCA treatment (Figure 4c). We also investigated multiple autophagy markers, such as Beclin1 and LC3B, due to the linked molecular pathways of autophagy and apoptosis [26]. In MCF-7 cells co-cultured with M1 macrophages, VCA augmented the levels of Beclin1 and LC3B, suggesting the induction of autophagy-dependent cell death, which may lead to apoptosis [27]. These results were confirmed through live/dead cell staining (Figure 4d).

### 2.5. Magnetic Levitation of Cells

**Figure 5:**
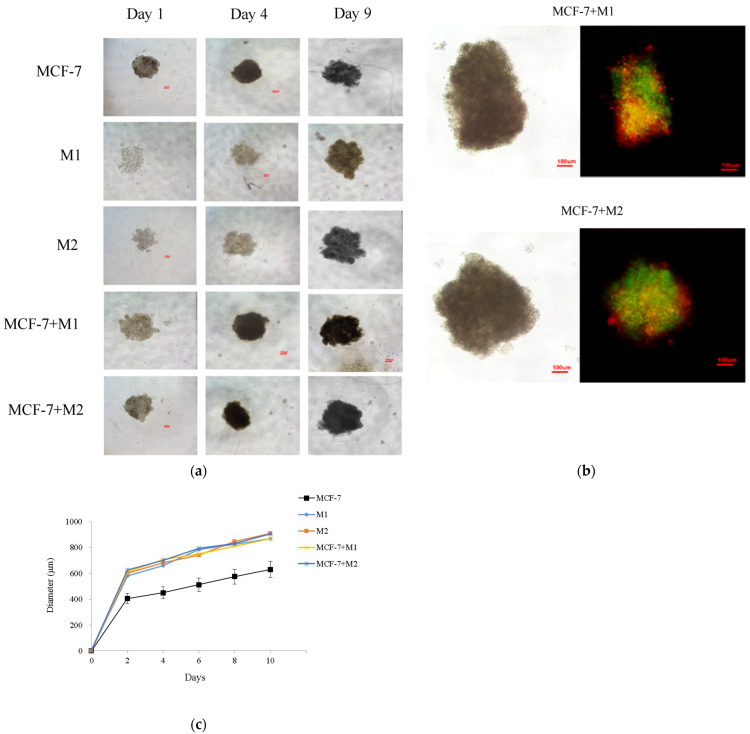
(**a**) Brightfield images of 3D cultures of MCF-7 and M1 and M2 macrophages from day 1 to day 10. (**b**) Spheroids with stained MCF-7 breast cancer cells (green) and macrophages (red) after 3 days of incubation; overlaid image shows the position of cell types throughout the spheroid. The scale bar is 100 μm for all images. (**c**) Plot of the diameter of day 1 to 10 grown 3D in vitro cells in 96-well plates. Scale bar = 100 μm. All experiments were performed in triplicate, with three independent repetitions.

We successfully levitated MCF-7 cells, and M1, and M2 macrophages into three-dimensional cultures, ensuring that the morphologies of all the cells remained unaffected throughout their binding to Nanoshuttle^TM^ (Figure 5a). Prior to their transformation into spheroids, the cells were labeled with a fluorescent cell tracker, allowing us to observe size changes over time and revealing a remodeled periphery (Figure 5c). While the normal M1 and M2 macrophages were able to form spheroids, the compactness increased slowly over time, resulting in an increase in spheroid size by day 9. Notably, NanoshuttleTM incubation and exposure to the magnetic field did not exert a noticeable effect on the spheroid development of MCF-7 cells and M1 and M2 macrophages.

### 2.6. 3D Co-Culture Assembly

Cells were incubated with Nanoshuttle^TM^ prior to their combination at a 1:4 ratio and levitated magnetically. On day 9, the co-culture cells had retained their integrity, demonstrating the successful assembly and maintenance of both MCF-7 and M1 or M2 macrophages. Following 12 h of levitation, MCF-7/M1 and MCF-7/M2 co-culture cells formed compact spherical structures that gradually increased in size. Subsequently, the resulting diameter of the cells was approximately 870 μm and 905 μm, respectively (Figure 5b).

### 2.7. Effect of VCA on 3D Cell Viability

**Figure 6:**
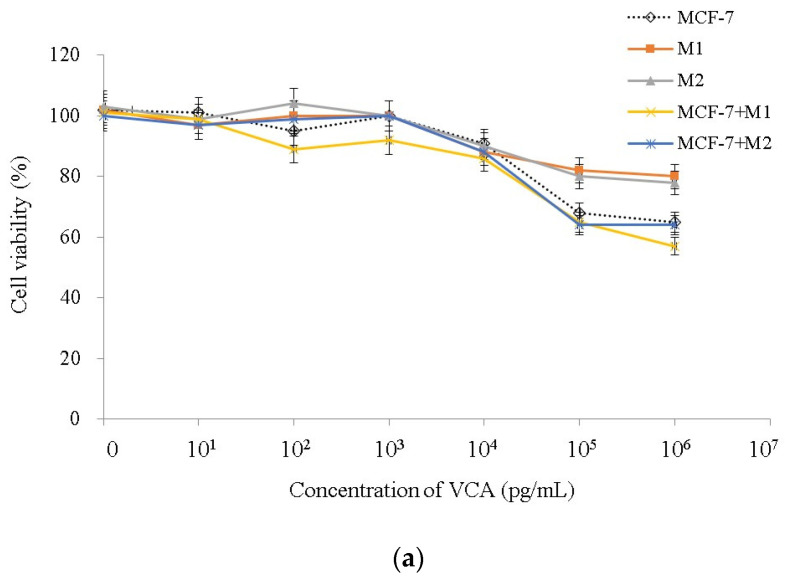

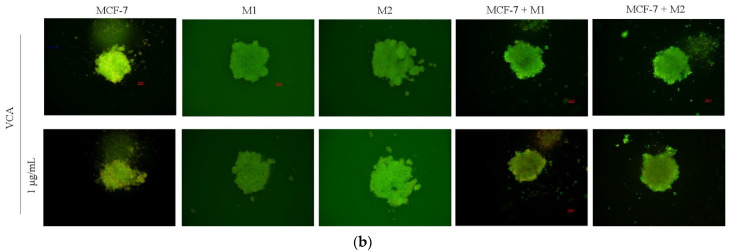
(**a**) A composite of three independent cell viability tests performed on MCF-7 cells and M1 and M2 macrophages in 3D cell culture. Cells were cultured in the absence or presence of different concentrations of VCA and the viability was measured by MTS assay. Results are expressed as means ± S.D (*n* = 3). (**b**) LIVE/DEAD cell assay was used with acridine orange (excitation 490 nm, emission 515 nm) and propidium iodide (PI, excitation 535 nm, emission 617 nm) with 1 μg/mL of VCA. Scale bar = 100 μm. All experiments were performed in triplicate, with three independent repetitions.

We utilized 3D cultures to evaluate the toxicity of VCA, exposing spheroids to various doses of VCA over a period of 10 days and measuring cell viability through MTS assay and live/dead cell staining. Significant effects were observed in MCF-7 cells at 1 μg/mL, while M1 and M2 macrophages showed more than 80% viability at the same dose. Therefore, we selected 1 μg/mL of VCA for our remaining experiments as it had no major effects on M1 and M2 macrophages when compared to the controls. We next assessed the effect of VCA on MCF-7 and M1 or M2 macrophage co-culture cells. Treatment with 1 μg/mL VCA in MCF-7/M1 co-culture cells resulted in a significant decrease in cell growth by 43 ± 1.41%, whereas monocultures of these cells exhibited 35 ± 2.95% growth inhibition for MCF-7 cells and 20 ± 3.77% for M1 macrophages at the same concentration. Additionally, cell viability was significantly reduced in MCF-7/M2 co-cultures after treatment with VCA (Figure 6a). Viable cells were detected through the LIVE/DEAD staining assay with spheroids, and these results were confirmed by the MTS assay. Imaging of the spheroids using acridine orange and propidium iodide allowed us to visualize the results under a fluorescence microscope, thereby confirming the viability assay results (Figure 6b).

### 2.8. The Impact of VCA on the Production of M1 and M2 Polarization Markers in 3D Co-Culture Cells

**Figure 7:**
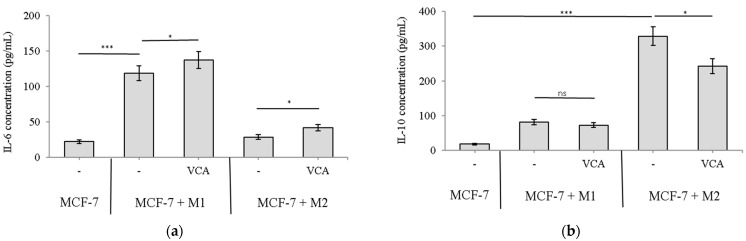
Determination of M1 and M2 macrophage markers in 3D MCF-7 co-cultures. M1 and M2 macrophages were co-cultured with MCF-7 cells to form 3D spheroids and treated with 1 μg/mL VCA for 10 days. (**a**) IL-6 and (**b**) IL-10 secretion in culture medium was measured by ELISA. Results are expressed as means ± S.D (*n* = 3). Statistical significance was determined by one-way ANOVA followed by Tukey’s post hoc test. * *p* < 0.05, *** *p* < 0.001, ns: not significant. All experiments were performed in triplicate, with three independent repetitions.

We assessed the protein secretions of M1 and M2 polarization markers through ELISA after 3D co-culture with MCF-7 cells, in the presence or absence of 1 μg/mL VCA for 10 days. The presence of VCA significantly affected the production of both M1 and M2 markers (Figure 7).

For the M1 marker IL-6 (Figure 7a), MCF-7 cells alone showed low levels of secretion (22.3 ± 2.6 pg/mL). Co-culture with M1 macrophages significantly increased IL-6 production (118.5 ± 10.7 pg/mL, *p* < 0.001 compared to MCF-7 alone). Notably, VCA treatment further enhanced IL-6 secretion in MCF-7 + M1 co-cultures (137.2 ± 11.8 pg/mL, *p* < 0.05 compared to untreated MCF-7 + M1). In MCF-7 + M2 co-cultures, IL-6 levels were initially low (28.7 ± 3.3 pg/mL), but VCA treatment induced a modest increase (41.5 ± 4.5 pg/mL, *p* < 0.05), suggesting a potential repolarization effect.

Regarding the M2 marker IL-10 (Figure 7b), MCF-7 cells alone produced minimal amounts (18.6 ± 2.3 pg/mL). MCF-7 + M1 co-cultures showed higher levels (81.3 ± 8.1 pg/mL), which were slightly reduced by VCA treatment (72.8 ± 7.1 pg/mL), although this reduction was not statistically significant (*p* > 0.05). In contrast, MCF-7 + M2 co-cultures exhibited significantly elevated IL-10 production (328.4 ± 26.5 pg/mL, *p* < 0.001 compared to MCF-7 alone). Importantly, VCA treatment substantially reduced IL-10 secretion in MCF-7 + M2 co-cultures (241.7 ± 21.5 pg/mL, *p* < 0.01 compared to untreated MCF-7 + M2), indicating a suppression of the M2 phenotype. Statistical significance was determined using one-way ANOVA followed by Tukey’s post hoc test.

These results demonstrate that VCA modulates macrophage polarization in the 3D tumor microenvironment, promoting M1-like characteristics while suppressing M2-associated factors. The trends observed in our 3D model are consistent with our 2D findings, albeit with some variations in magnitude. This suggests that the 3D model effectively replicates key aspects of the 2D system while potentially capturing additional complexities of the tumor microenvironment, such as diffusion gradients and more intricate cell–cell interactions. The less pronounced differences in some conditions compared to 2D cultures may be attributed to these additional factors in the 3D model, highlighting the importance of considering spatial organization in studying tumor–macrophage interactions.

## 3. Discussion

The microenvironment of breast tumors dramatically influences tumor development and responses to therapeutic interventions. Consequently, there is a growing focus on investigating innovative treatments that target the microenvironment in relation to invasive and metastatic progression [4]. Various cell types such as macrophages, fibroblasts, and adipocytes constitute the breast tumor stroma [28,29]. The incorporation of these extra cells into an in vitro model may alter the cell–cell interactions and signaling similar to tumor microenvironments [30]. Many studies have demonstrated that in cancerous tumors, macrophages frequently demonstrate M2-like behavior. M2 macrophages associated with tumors assume a critical position in tumor cell growth, sustaining vitality, and initiating processes such as angiogenesis and metastasis [31]. In contrast, M1 macrophages possess cytotoxic properties against both pathogens and tumor cells. This dichotomy between M1 and M2 macrophages plays a crucial role in shaping the tumor microenvironment and influencing cancer progression.

In this study, we successfully established a model of macrophage polarization using human THP-1 monocytes. We differentiated these cells into macrophages using 10 ng/mL PMA, followed by a 3-day resting period. To induce M1 polarization, we treated cells with 20 ng/mL IFN-γ and 100 ng/mL LPS, while M2 polarization was achieved using 20 ng/mL each of IL-4 and IL-13. Several classical M1 markers, including IL-6 and TNF-α, were measured with ELISA to reveal a significant increase in pro-inflammatory marker secretion. For M2 macrophages, the protein levels of M2 markers, such as IL-10 and CCL18, were investigated. In particular, CCL18 is recognized as an indicator of M2 macrophages and can be induced by IL-4, IL-10, and IL-13 [10]. The secretion of both IL-10 and CCL18 was augmented, yet no release of any M1 macrophage markers was observed in M2-polarized macrophages, consistent with previous reports [10,14,32].

To further validate our macrophage polarization protocol, we conducted flow cytometry analysis (Figure 1e–l). The distinct expression patterns of surface markers between M1 and M2 macrophages confirmed the successful differentiation of THP-1 monocytes into these two phenotypes. M1 macrophages showed a significant increase in CD80 and CD86 expression, which are key markers of pro-inflammatory macrophages [33]. In contrast, M2 macrophages exhibited elevated levels of CD163 and CD206, consistent with their anti-inflammatory and tissue-repair functions [10]. The time-course analysis revealed that these phenotypic changes were stable over the experimental period, ensuring the reliability of our co-culture experiments. Importantly, the flow cytometry data complemented our ELISA results, providing a comprehensive characterization of our M1 and M2 macrophage populations [34]. This robust validation of our macrophage model strengthens the foundation for our subsequent investigations into the interactions between these distinct macrophage phenotypes, breast cancer cells, and VCA.

To investigate the interplay between macrophages, breast cancer cells, and VCA, we employed a Transwell^®^ co-culture system. This setup allowed us to examine the indirect effects of M1 and M2 THP-1 macrophages on MCF-7 breast cancer cells in response to VCA treatment. Incubating pro-inflammatory M1 macrophages with MCF-7 cells in the presence of VCA led to a significant increase in IL-6 and TNF-α mRNA expression (Figure 3a,b). Interestingly, regarding M2 markers, the expressions of IL-10 and CCL18 were strongly suppressed in cells incubated with VCA (Figure 3c,d). These findings suggest that VCA may have the potential to modulate macrophage phenotypes within the tumor microenvironment.

Many anti-cancer agents have demonstrated the ability to counteract the tendency of cancer cells to induce a shift in macrophages towards the M2 phenotype. For instance, metformin activates AMPK-NF-κB signaling in breast cancer cells, ultimately regulating the balance of M1/M2 expression [35]. The induction of breast cancer cell apoptosis by etoposide was enhanced in cells co-cultured with M1 macrophages [14]. Furthermore, paclitaxel reprogrammed M2-polarized macrophages into an M1-like phenotype in a TLR-4-dependent manner in both in vitro and in vivo models [36]. Our results with VCA align with these studies, suggesting that exploring the potential strategy of using VCA to convert M2 macrophages into an M1 phenotype holds promise in the field of breast cancer research. This approach could potentially enhance the anti-tumor immune response and improve the efficacy of breast cancer treatments.

To further elucidate the mechanisms by which VCA affects breast cancer cells in the context of macrophage co-culture, we conducted a series of apoptosis-related experiments. A flow cytometry study using Annexin V/Propidium iodide staining was performed to detect apoptosis in cells. VCA increased apoptosis in both MCF-7 cells co-cultured with M1 macrophages and MCF-7 cells co-cultured with M2 macrophages. Notably, the pro-apoptotic effect of VCA was observed in both co-culture conditions, suggesting robust anti-cancer activity regardless of the macrophage phenotype present. Since several reports have demonstrated that changes in mitochondrial structure and mitochondrial membrane potential can initiate nuclear features of apoptosis [37], we employed the MuseTM MitoPotential Kit to assess changes in mitochondrial membrane potential in MCF-7 cells. An increase in depolarized dead cells was observed in both MCF-7 cells co-cultured with M1 or M2 macrophages when treated with VCA. This finding indicates that VCA-induced apoptosis may be mediated, at least in part, through mitochondrial dysfunction. To gain further insights into how secreted factors from the co-cultured macrophages might influence apoptosis and autophagy, we conducted protein extraction from the MCF-7 cells. Western blotting analysis was carried out to measure apoptosis-related proteins, including Bcl-2, Bax, and caspase-3. Upon treatment with VCA, there was an increase in the abundance of Bax and cleaved caspase-3, along with a decrease in Bcl-2 in MCF-7 cells incubated alongside M1 macrophages, as compared to control cells. These changes in protein expression are consistent with the activation of the intrinsic apoptosis pathway. Additionally, VCA significantly elevated the percentage of apoptotic and depolarized cells in MCF-7/M1 co-culture cells, in contrast to control cells incubated without VCA. Collectively, these results demonstrate that VCA enhances apoptosis in breast cancer cells, particularly in the presence of M1 macrophages, suggesting a potential synergistic effect between VCA and the pro-inflammatory tumor microenvironment.

Autophagy, which is a lysosomal breakdown process, plays a critical role in eliminating and recycling defective organelles, thereby maintaining cellular integrity. Previous studies have indicated that autophagy can promote tumor cell survival by shielding cells from oxygen deprivation, malnutrition, oxidative damage, and by generating chemo-resistance in cancer [38,39]. However, the role of autophagy in cancer is complex and context-dependent. Levine et al. reported that Beclin1 suppresses cell proliferation and tumorigenesis in both in vivo and in vitro settings, thus inhibiting tumor growth. Therefore, downregulating autophagy may contribute to the progression of breast cancer [40]. Additionally, LC3B is regarded as a reliable indicator of autophagy in cancer cells since it is a key component in the generation of autophagosomes [41]. In our study, we investigated the potential role of autophagy in VCA-mediated effects on breast cancer cells. We observed a significant increase in the levels of Beclin1 and LC3B in MCF-7 cells co-cultured with M1 macrophages following VCA treatment, compared to control cells. This upregulation of autophagy markers suggests that VCA may modulate autophagic processes in breast cancer cells, particularly in the context of a pro-inflammatory microenvironment. Given that various anti-cancer agents induce autophagy-dependent cell death, such as tamoxifen [42,43], it is plausible that VCA might excessively activate autophagy, potentially leading to apoptosis. This “autophagic cell death” mechanism could represent an additional pathway through which VCA exerts its anti-cancer effects, complementing its pro-apoptotic activities. Further investigation into the interplay between VCA-induced autophagy and apoptosis in breast cancer cells, especially in the context of macrophage co-culture, may provide valuable insights into the compound’s therapeutic potential.

Our findings reveal that M1 and M2 macrophages, derived from THP-1 monocytes, can influence the immune and apoptotic responses of MCF-7 breast cancer cells to VCA. In the 2D co-culture model, M1 macrophages exhibited a cytotoxic effect and enhanced the induction of apoptosis by VCA. In addition, although M2 macrophages had a protective effect and reduced apoptosis in breast cancer cells, exposure to VCA effectively reversed this protective effect. These results underscore the importance of considering the tumor microenvironment, particularly the presence and phenotype of tumor-associated macrophages, when evaluating the efficacy of anti-cancer compounds like VCA.

While 2D cell cultures are commonly employed to assess optimal therapeutic doses before testing in animal cancer models, they fall short of fully replicating the complexity of the intricate tumor microenvironment [44]. A significant gap exists between the knowledge gained from two-dimensional in vitro models and in vivo studies, with data from 2D models often inadequately predicting the magnitude of therapeutic efficacy in vivo [45]. One reason for this discrepancy is that in vivo cells are organized into three-dimensional structures that do not adhere to flat surfaces. Recognizing these limitations, three-dimensional in vitro models have been developed to bridge the gap between 2D systems and animal studies [46]. To address this issue in our study and to more accurately model the tumor microenvironment, we employed a 3D co-culture system using magnetic levitation. This approach allowed us to investigate the interactions between breast cancer cells, macrophages, and VCA in a more physiologically relevant context.

In this study, we employed spheroid models, a common strategy in breast cancer research, as these cells tend to cluster without adhering to exterior surfaces [44]. Previous studies have introduced an innovative in vitro model that utilizes a magnetic lifting device under standard culture conditions to generate 3D structures that mimic in vivo tissues [44,47,48,49]. We adapted this system to simulate heterogeneous breast tumors without using scaffolds, enabling cell–cell interactions. Specifically, we co-cultured MCF-7 breast cancer cells with M1 or M2 macrophages and subjected them to magnetic levitation. We tracked the diameter from day 1 to 10 to physically characterize the formation of an in vitro breast tumor in three dimensions, using breast cancer and macrophage cells. Microscopic images of these cells were analyzed, revealing the development of dense and thick 3D cultures. To evaluate the impact of VCA treatment, we assessed cell viability using the MTS assay and compared the results with the 2D approach.

Notably, VCA’s effects on viability were significant in both 2D and 3D systems. This consistency across different culture models strengthens the validity of our findings. Jaganathan et al. reported that doxorubicin and Doxil^®^ did not significantly reduce cell survival in in vitro 3D tumors compared to their effect on 2D cultures, which was attributed to poor drug penetration [44]. This restricted penetration was also observed in tightly packed in vivo tumors [50,51]. In contrast, our three-dimensional in vitro breast tumor model generated through magnetic levitation appeared capable of replicating the physical barriers akin to those encountered in vivo, without hindering VCA transport. This suggests that VCA may have favorable penetration properties, which could be advantageous for potential therapeutic applications.

Moreover, in the 3D co-culture setting, VCA treatment showed a trend towards increased secretion of the M1 marker IL-6 in MCF-7 + M1 co-cultures, although this increase was not statistically significant. Conversely, VCA significantly suppressed the M2 marker IL-10 in MCF-7 + M2 co-cultures (Figure 7). These results indicate that VCA’s ability to modulate macrophage phenotypes is preserved in more complex 3D environments, albeit with some variations in magnitude compared to the 2D model. It is worth noting that while we examined both IL-10 and CCL18 as M2 markers in our 2D experiments, only IL-10 was measured in the 3D model, which limits direct comparison between the two systems for all markers.

It is important to note that while our study provides comprehensive insights into the effects of VCA on apoptosis and macrophage polarization, we were unable to include Ki67 staining results as initially suggested by the reviewers. Ki67 is a widely used marker for cellular proliferation [52], and its inclusion could have provided additional information about VCA’s effects on cancer cell growth [53]. This limitation in our study presents an opportunity for future research to further elucidate the impact of VCA on breast cancer cell proliferation in the context of macrophage co-culture. Despite this limitation, our current findings on apoptosis, mitochondrial membrane potential, and macrophage polarization offer valuable insights into VCA’s potential as an anti-cancer agent. Another limitation of this study is the lack of TNF-α and CCL18 measurements for M1 and M2 polarization, respectively, in the 3D model. This restricts direct comparisons of all markers between the 2D and 3D systems. Future studies addressing this limitation could provide a more comprehensive analysis of macrophage behavior and polarization in different culture environments. Also, while the current study provides valuable insights into VCA’s effects on breast cancer cells and macrophages in 2D and 3D in vitro models, further validation in animal models would be beneficial. Future in vivo studies could assess the impact of VCA on tumor growth and immune cell function, potentially bridging the gap between these in vitro findings and clinical applications.

In summary, our findings highlight the capacity of VCA to influence M1 and M2 polarization in breast cancer cells. Furthermore, we demonstrate that VCA exerts anti-cancer effects and modulates macrophage phenotypes in both 2D and 3D culture systems. Our utilization of magnetic 3D cell cultures offers promising reproducibility similar to traditional 2D cultures, potentially bridging the gap between 2D models and animal studies. While this study provides insights into VCA’s effects on breast cancer cells and macrophages, further investigation into the underlying molecular mechanisms could enhance our understanding. Future research could explore signaling pathway regulations and changes in protein expression profiles induced by VCA treatment. Such mechanistic studies could potentially reveal novel targets and pathways involved in VCA’s anti-cancer and immunomodulatory effects, thereby expanding our knowledge of its therapeutic potential. Notably, our study represents a pioneering effort, being the first to provide empirical evidence of VCA’s divergent effects on M1 and M2 macrophages co-cultured alongside breast cancer cells. These insights contribute to our understanding of how natural compounds like VCA can potentially be leveraged to modulate the tumor microenvironment and enhance anti-cancer responses.

## 4. Materials and Methods

### 4.1. Purification of VCA

Korean mistletoe was gathered from oak trees in Kangwon-do, Republic of Korea. The botanical verification was performed by Professor Jon-Suk Lee from Seoul Women’s University, Republic of Korea, and a voucher specimen (VCA101) was deposited at the College of Pharmacy, Sunchon National University, Republic of Korea. The isolation of lectin followed previously established methods. In brief, SP Sephadex C-50 (Amersham Pharmacia Biotech Inc., Stockholm, Sweden) and asialofetuin (Sigma, St. Louis, MO, USA)-Sepharose 4B were utilized for lectin isolation, while ultrafiltration (Millipore, Billerica, MA, USA) was employed for concentration. We also measured the protein concentration using the Bio-Rad protein assay kit (Bio-Rad, Hercules, CA, USA). The sodium dodecyl sulfate-polyacrylamide gel electrophoresis (SDS-PAGE) technique was utilized to assess both molecular weight and purity.

### 4.2. Cell Culture and Reagents

Human breast cancer cells (MCF-7) and human monocytic THP-1 cells were obtained from the Republic of Korea Cell Line Bank (KCLB, Seoul, Republic of Korea). The cells were cultured in RPMI 1640 medium supplemented with the following: 10% (*v*/*v*) fetal bovine serum (FBS, Life Technologies, Carlsbad, CA, USA), 1% (*v*/*v*) non-essential amino acids, 1% (*v*/*v*) L-glutamine, and 1% (*v*/*v*) penicillin–streptomycin (Life Technologies, Carlsbad, CA, USA). The cultures were maintained at 37 °C under a 5% CO_2_-saturated atmosphere. We utilized the following commercially available antibodies and reagents: caspase-3, cleaved caspase-3, Bcl-2, Bax, LC3B, Beclin-1, β-actin, anti-rabbit IgG, horseradish peroxidase (HRP)-linked antibody, and the LumiGLO^®^ chemiluminescent detection system, all from Cell Signaling Technology (Danvers, MA, USA).

### 4.3. THP-1 Macrophage Differentiation and Polarization

THP-1 monocytes were differentiated into macrophages by treatment with 10 ng/mL phorbol 12-myristate-13-acetate (PMA) in 24-well cell culture plates for 48 h. After differentiation, cells were washed twice with culture medium and rested for 24 h to reach the M0 macrophage state.

To induce M1 polarization, M0 macrophages were stimulated with culture medium containing 20 ng/mL IFN-γ and 100 ng/mL LPS for 24 h. For M2 polarization, M0 macrophages were stimulated with 20 ng/mL IL-4 and 20 ng/mL IL-13 for 72 h.

To characterize and confirm macrophage differentiation and polarization, flow cytometry analysis was performed at four key timepoints: Day 0 (undifferentiated THP-1), Day 2 (after PMA treatment), Day 3 (24 h after polarization stimuli), and Day 5 (72 h after M2 polarization stimulus). Cells were harvested, washed with PBS containing 1% BSA, and incubated with fluorochrome-conjugated antibodies against CD80 and CD86 (M1 markers), and CD163 and CD206 (M2 markers) for 30 min at 4 °C in the dark. After washing, cells were analyzed using a FACSCalibur flow cytometer (BD Biosciences, San Jose, CA, USA). Data were analyzed using FlowJo software v10.9 (Tree Star, Ashland, OR, USA).

The expression of phenotype-specific cytokines was confirmed using ELISA. Culture supernatants were collected at the end of the polarization period (24 h for M1, 72 h for M2) and analyzed for IL-6, TNF-α (M1 markers), IL-10, and CCL18 (M2 markers) using commercial ELISA kits (R&D Systems, Minneapolis, MN, USA) according to the manufacturer’s instructions.

### 4.4. 3D Co-Culture Using Transwell

**Figure 8:**
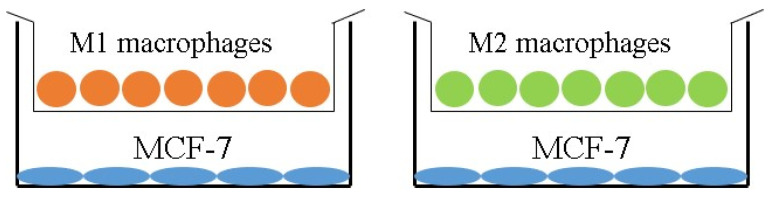
Two-dimensional co-culture model using Transwell. MCF-7 cells were plated in the lower chamber and M1 or M2 macrophages were seeded in the upper chamber and co-cultured in Transwell before being incubated with or without VCA for 24 h.

For the 2D co-culture experiment, MCF-7 cells were seeded in the lower chamber of Transwell inserts (BD Biosciences, San Jose, CA, USA). M1 or M2 macrophages were then added to the upper chamber and co-cultured for 24 h. Following this initial co-culture period, VCA was added to both chambers at various concentrations and incubated for an additional 24 h. After treatment, we assayed MCF-7 cells in the lower chamber and collected the supernatant from both chambers for further analysis, as illustrated in Figure 8.

### 4.5. Cell Viability Assay

Using the 3-(4,5-Dimethylthiazol-2-yl)-2,5-diphenyltetrazolium bromide (MTT) assay, we assessed cell viability in 2D cell cultures by exposing the cells to varying concentrations of VCA at 37 °C in a humidified atmosphere with 5% CO_2_. Cells were exposed to various concentrations of VCA at 37 °C under humidified and 5% CO_2_ atmospheric conditions, and reacted with 50 μL of MTT dye reagent for 4 h. Subsequently, the MTT-containing solution was removed and formazan precipitates were solubilized using 200 μL of dimethyl sulfoxide (DMSO). We then employed a microplate reader (Sunrise^®^, Tecan, Männedorf, Switzerland) to measure the absorbance at 570 nm. For 3D cell cultures, we used 3-(4,5-dimethylthiazol-2-yl)-5-(3-carboxymethyoxyphenyl)-2-(4-sulfophenyl)-2H-tetrazolium, inner salt (MTS, Promega, Southampton, UK) and determined the formazan production by measuring the absorbance at 450 nm using the same microplate reader (Sunrise^®^, Tecan, Männedorf, Switzerland).

### 4.6. Live/Dead Cell Staining

To verify cell viability, we employed the LIVE/DEAD viability/cytotoxicity kit (Invitrogen, Waltham, MA, USA). This was done in conjunction with acridine orange (excitation 490 nm, emission 515 nm) and propidium iodide (PI; excitation 535 nm, emission 617 nm), after cultivating cells over a span of 24 h along with varying doses of VCA. Living cells possess a characteristic of retaining acridine orange, which results in even fluorescence throughout their structure. Contrarily, PI acts as a nuclear staining dye and faces barriers when attempting to pass through viable cell membranes. When a cell dies, PI penetrates the nucleus through disrupted regions, binding to the DNA double helix within the cell and emitting red fluorescence. We simultaneously monitored viable and dead cells using fluorescent microscopy (Nikon, Kōnan, Japan).

### 4.7. Quantitative Real-Time Polymerase Chain Reaction (qPCR)

Using the RNeasy mini kit (Qiagen, Hilden, Germany), we extracted total RNA from cells; then, we synthesized cDNA with a cDNA synthesis kit (Qiagen, Hilden, Germany) from 1 μg of RNA. Subsequently, for qPCR, we employed the Quanti Nova SYBR Green PCR Kit (Qiagen, Hilden, Germany) and the ECO real-time PCR system (Illumina Inc., San Diego, CA, USA). The following primer sequences were used: Human IL-6 (forward 5′-ATGAACTCCTTCTCCACAAGCGC-3′, reverse 5′-GAAGAGCCCTCAGGCTGGACTG-3′); Human IL-10 (forward 5′-GCTGTCATCGATTTCTTCCC-3′, reverse 5′-CTCATGGCTTTGTAGATGCCT-3′), human TNF-α (forward 5′-CGAGTGACAAGCCTGTAGCC-3′, reverse 5′-TGAAGAGGACCTGGGAGTAGAA-3′), Human CCL18 (forward 5′-CTTTCCCCTTTCCCTTCAAC-3′, reverse 5′-GTGCTGAGCAAAACCATTCA-3′), and beta-actin (forward 5′-GGAAATCGTGCGTGACATT-3′, reverse 5′-CAGGCAGCTCGTAGCTCTT-3′).

### 4.8. Enzyme-Linked Immunosorbent Assay (ELISA)

In the ELISA procedure, we utilized 96-well microplates, coating them overnight with capture antibodies at 4 °C to assess cytokine secretions. Following the addition of a blocking buffer composed of 1% BSA in PBS to prevent non-specific binding, the plates were incubated for 1 h and subsequently washed three times with a washing buffer comprising PBS and 0.5% Tween 20. We added samples and standards to the plates and allowed them to incubate for 2 additional h. Subsequently, we washed the plates five times before subjecting them to the working detector for 1 h and performing a further five washes. Afterward, the substrate solution was added, and the plate was incubated in the dark for 30 min. We stopped the reaction by adding a stop solution (1 M phosphoric acid) and measured color development using a Sunrise^®^ microplate reader (Tecan, Männedorf, Switzerland) at 450 nm.

### 4.9. Apoptosis Analysis

We quantified apoptotic cells after a 24 h treatment with VCA, following the guidelines provided by the manufacturer for the Muse Annexin V and Dead Cell Kit (Millipore, Billerica, MA, USA). During this process, we combined the cell suspensions with the Muse Annexin V and Dead Cell Kit reagent and incubated it for 20 min at room temperature in the dark. To calculate and analyze apoptotic cells, we used the Muse Cell Analyzer (Millipore, Billerica, MA, USA).

### 4.10. Mitopotential Assay

After a 24 h VCA treatment, we collected 100 μL of cell suspensions for the mitopotential assay. The MuseTM MitoPotential assay enables the simultaneous measurement of changes in mitochondrial potential (commonly recognized as an early characteristic of apoptosis and cellular stress) and the permeabilization of the cellular plasma membrane or cell death. This assay uses a mitopotential dye with cationic properties and lipophilicity to detect variations in mitochondrial membrane potential, in combination with 7-aminoactinomycin D (7-ADD) as an indicator of cell death. We added MitoPotential working solution (95 μL) to the cell suspension and mixed it by pipetting. Afterward, the cells were incubated at 37 °C in a CO_2_ incubator for 20 min. Subsequently, we added and mixed 5 μL of Muse MitoPotential 7-ADD reagent and incubated the mixture at room temperature for 5 min. The cells were analyzed for changes in mitochondrial membrane potential using the Muse Cell Analyzer by Milipore (Billerica, MA, USA).

### 4.11. Western Blotting

To perform Western blotting, we initiated the process by washing the cells with PBS. Following this, we added 1 mL of Radioimmunoprecipitation Assay (RIPA) buffer for cell lysis. After centrifuging the cell lysate at 8000× *g* for 5 min, we loaded it onto an SDS-PAGE gel. Subsequently, proteins were transferred to a polyvinylidene fluoride membrane and washed with 25 mL of tris-buffered saline (TBS, 50 mM Tris-Cl, 150 mM NaCl, pH 7.5) for 5 min. The membrane was then incubated with a blocking buffer (5% *w*/*v* BSA) for 1 h at room temperature, followed by three 5 min washes in TBS-T (TBS with 0.1% Tween 20). After this, the membrane was incubated with the primary antibody in TBS-T with 5% BSA. Washing steps were repeated using TBS-T, and the membrane was subsequently incubated with an HRP-conjugated secondary antibody for 1 h. Finally, after three additional 5 min washes with TBS-T, we detected the protein using LumiGLO^®^ (Cell Signaling Technology, Danvers, MA, USA).

### 4.12. Magnetic Levitation for 3D In Vitro Tumor Model

The 3D cultures were formed using the Bio-Assembler Kit (Nano3D Biosciences, Houston, TX, USA) as previously reported [47]. In short, NanoShuttleTM was added to flasks of MCF-7, M1, and M2 macrophages grown in 2D at 80% confluence and incubated overnight. Using trypsin, we detached the cells and resuspended them in the culture medium. Subsequently, we added this resulting cell suspension to both 24- and 96-well ultra-low attachment plates (Corning, Tewksbury, MA, USA). Upon placing a magnetic driver with a field strength of 50 G, the cells were levitated to the air–medium interface. These levitated cells were then incubated at 37 °C in a humidified atmosphere with 5% CO_2_ overnight in an incubator.

### 4.13. 3D Co-Culture Assembly

We created 3D co-cultures of MCF-7 cells with either M1 or M2 macrophages using the magnetic levitation method. After an overnight incubation with NanoShuttleTM, we levitated 5 × 10^5^ cells of each type into the 3D cultures. To construct the 3D co-culture, we used a magnetic pen and cylinder insert to lift the MCF-7 and M1/M2 3D cultures for 4 h. These co-cultures were then submerged in culture medium within either a 24- or 96-well ultra-low attachment plate (Corning, Tewksbury, MA, USA) for an additional 4 h. A magnetic driver was placed on top of the lid to facilitate the ascent of the cells.

### 4.14. Imaging and Measurement of 3D Spheroids

We used an Olympus IX53 microscope (Tokyo, Japan) equipped with an Insight camera to capture Brightfield images of the spheroids over a 10-day period. Subsequent image analysis was performed using (NIH, Bethesda, MD, USA). Prior to spheroid formation, we labeled MCF-7 breast cancer cells and macrophages with green and red cell trackers (Promega, Southampton, UK), respectively. All images are representative and include a scale bar of 100 μm.

### 4.15. Statistical Analysis

All experiments were conducted in triplicate, and the results were presented as means ± standard deviation (S.D.). Statistical analysis of the data was performed using GraphPad software (version 7.00). For comparisons between two groups, Student’s *t*-test was used. For comparisons among multiple groups, one-way ANOVA followed by Tukey’s post hoc test was employed. Statistical significance was defined as *p* < 0.05, *p* < 0.01, and *p* < 0.001.

## Data Availability

Data are contained within the article.
